# Biodiversidata: A novel dataset for the vascular plant species diversity in Uruguay

**DOI:** 10.3897/BDJ.8.e56850

**Published:** 2020-10-26

**Authors:** Florencia Grattarola, Andrés González, Patricia Mai, Laura Cappuccio, César Fagúndez-Pachón, Florencia Rossi, Franco Teixeira de Mello, Lucía Urtado, Daniel Pincheira-Donoso

**Affiliations:** 1 School of Life Sciences, University of Lincoln, Lincoln, United Kingdom School of Life Sciences, University of Lincoln Lincoln United Kingdom; 2 Museo Nacional de Historia Natural, Montevideo, Uruguay Museo Nacional de Historia Natural Montevideo Uruguay; 3 Departamento de Ecología y Gestión Ambiental, Centro Universitario Regional del Este (CURE), Universidad de la República, Maldonado, Uruguay Departamento de Ecología y Gestión Ambiental, Centro Universitario Regional del Este (CURE), Universidad de la República Maldonado Uruguay; 4 Departamento Interdisciplinario de Sistemas Costeros y Marinos, Centro Universitario Regional del Este (CURE), Universidad de la República, Rocha, Uruguay Departamento Interdisciplinario de Sistemas Costeros y Marinos, Centro Universitario Regional del Este (CURE), Universidad de la República Rocha Uruguay; 5 MacroBiodiversity Lab, School of Biological Sciences, Queen’s University Belfast, Belfast, United Kingdom MacroBiodiversity Lab, School of Biological Sciences, Queen’s University Belfast Belfast United Kingdom

**Keywords:** Species occurrence records, biodiversity data gaps, data mobilisation, Tracheophyta, Río de la Plata grasslands, South America, Uruguay

## Abstract

**Background:**

South America hosts some of the world’s most prominent biodiversity hotspots. Yet, Uruguay – a country where multiple major ecosystems converge – ranks amongst the countries with the lowest levels of available digital biodiversity data in the continent. Such prevalent data scarcity has significantly undermined our ability to progress towards evidence-based conservation actions – a critical limitation for a country with a strong focus on agricultural industries and only 1.3% of the land surface guarded by protected areas. Under today’s rapid biodiversity loss and environmental changes, the need for open-access biodiversity data is more pressing than ever before. To address this national issue, Biodiversidata – Uruguay’s first Consortium of Biodiversity Data – has recently emerged with the aim of assembling a constantly growing database for the biodiversity of this country. While the first phase of the project targeted vertebrate biodiversity, the second phase presented in this paper spans the biodiversity of plants.

**New information:**

As part of the second phase of the Biodiversidata initiative, we present the first comprehensive open-access species-level database of the vascular plant diversity recorded in Uruguay to date (i.e. all species for which data are currently available and species presence has been confirmed). It contains 12,470 occurrence records from across 1,648 species and 160 families, which roughly represents 60% of the total recorded flora of Uruguay. The primary biodiversity data include extant native and introduced species from the lycophytes, ferns, gymnosperms and angiosperms groups. Records were collated from multiple sources, including data available in peer-reviewed scientific literature, institutional scientific collections and datasets contributed by members of the Biodiversidata initiative. The complete database can be accessed at the Zenodo repository: doi.org/10.5281/zenodo.3954406

## Introduction

South America stands out as one of the planet’s regions with the highest levels of species-richness and endemisms (Myers 2003, Myers et al. 2000). Within this subcontinent, Uruguay occupies an area characterised by a high floristic diversity in one of the most extensive temperate grasslands on the globe (Soriano et al. 1992, Andrade et al. 2018). This country embodies a transitional territory where multiple floristic elements of diverse origin converge (Chebataroff 1942, Chebataroff 1960, Brussa and Grela 2007, Grela 2004). However, the boundaries and ecotones of these phytogeographic regions have remained under sustained debate fundamentally given the lack of comprehensive and accessible databases on the diversity and distribution of species from Uruguay. While recent efforts have endeavoured to resolve this limitation for vertebrates (Grattarola et al. 2019a), data on the country’s plant biodiversity remain limited and scattered across a range of small-scale databases, the majority of which are inaccessible. Our work fills this gap by presenting such a comprehensive database for the plant biodiversity of Uruguay.

The compilation of georeferenced plant data is a relatively-recent practice in Uruguay (Brazeiro et al. 2008, Grela 2004, Haretche et al. 2012). In all cases, maps have been created, based on lists of species found within individual grid-cells of 660 km^2^ size, without specific information about their actual locations or when the species were observed or collected. This method is intrinsically affected by a serious loss of spatial and temporal information (i.e. data leakage, Peterson et al. 2018). Yet, until today, it has been the standard approach to build national species distribution databases. It is imperative for Uruguay to change its biodiversity information management to a widespread approach, based on the digitisation of specimen and literature records and on open-access of the available databases. Under accelerating scenarios of human-induced alterations of the global climate and natural landscapes (Butchart et al. 2010, Lovejoy and Hannah 2019, Parmesan 2006), with more than 36.2% of the Uruguayan territory already modified (Brazeiro et al. 2020, Cespedes-Payret et al. 2009), the least developed network of protected areas in the region (Soutullo and Gudynas 2006) and the lowest levels of digitally-available biodiversity data of Latin America (Grattarola et al. 2019a), developing scientific databases in Uruguay is a critical need.

### The Biodiversidata Initiative

Biodiversidata – Uruguay’s first Consortium of Biodiversity Data (https://biodiversidata.org/) – has recently emerged with the aim of assembling a constantly growing, open-access database for Uruguay’s biodiversity (Grattarola et al. 2019a). The range of beneficiaries of the biodiversity data resources that this initiative provides is wide, including individuals and institutions from the scientific, educational and governmental sectors. Biodiversidata relies on the assemblage of biodiversity experts, with the aim of collating a comprehensive database spanning all components of the country’s biodiversity (Grattarola et al. 2019a). This aim is being achieved by overcoming the main obstacles detected in the process of data-sharing (Grattarola and Pincheira-Donoso 2019), focusing on data digitisation, curation and standardisation, as well as on the use of the data to collaboratively address questions of conceptual global impact/interest. The data collated for the tetrapod vertebrates have enabled us to understand that most of the country remains neglected by scientific efforts, while a few areas have historically been consistently sampled. In this second stage, we focused on plants. The sampling bias scenario seems to mirror the scenario described for tetrapods (Grattarola et al. 2019a). Here, we present the first comprehensive open-access database of vascular plant species of Uruguay, including all species for which data are currently available. The total number of records collated is 12,470, which includes 1,648 species out of the 3,000 species that have been reported for this country (including records to be confirmed) (Marchesi et al. 2013, Zuloaga et al. 2019)(Table 1). Combined with the first phase that presented a database for tetrapods (Grattarola et al. 2019b), this current expansion of Biodiversidata to plant biodiversity provides an unprecedented resource, anticipated to have a major impact on the development of biodiversity studies and management in Uruguay.

## Sampling methods

### Sampling description

The primary data were collated from a range of different sources such as online databases, field guides, reports and primary literature, as well as Biodiversidata members’ original field/herbarium records. A complete list of sources for the occurrence records is shown in Table 2. Regardless of the source, the data collated aimed to include information on the record event: collection date when available, geographic location and taxonomic identification amongst others. The majority of the records were standardised following the Darwin Core Biodiversity Data Standard (Wieczorek et al. 2012) in line with FAIR (Findable, Accessible, Interoperable and Reusable) data Principles (Wilkinson et al. 2016); see a complete description of terms in the section Data Description. R software (R Core Team 2020) was used to automate and batch process the data cleaning procedure and visualisation analyses. The scripts used can be accessed at our GitHub repository github.com/bienflorencia/rBiodiversidata.

The data from bibliographic references were obtained from searches based on the use of more than 30 sources which were largely heterogeneous in the amount of information available for each record. The information about the source was captured for each record using the ‘associatedReferences’ Darwin Core term. The data extracted consisted of taxa names, their geographic location and date of the collection/observation event when available, as well as information about collectors and identifiers. In some cases, georeferencing of the point locations was needed and relevant information was captured under the terms ‘coordinateUncertaintyInMeters’, ‘coordinatePrecision’ and ‘georeferenceRemarks’ (see more details in Steps description subsection).

The data from online sources were accessed through GBIF via ‘rgbif’ (Chamberlain et al. 2020a), using the following search parameters: 1) Tracheophyta as taxon, 2) 'UY' in the country code (= Uruguay), 3) year of collection from 1900 onwards, 4) with geographic coordinates and no spatial issues associated and 5) including data of ‘Preserved Specimen’ and ‘Human Observation’ categories. The chosen parameters were considered to reduce the data cleaning time, given the purpose of use of the data being collected and the limited timeline of the project under course. Records with 'LivingSpecimen' or 'FossilSpecimen' as the basis of records were filtered to avoid crop/cultivated and extinct species. As a perspective, records with 'Unknown' base should be checked in the future. As well, the date lower limit and the constraint of records with coordinates and no spatial issues associated were selected to minimise potential taxonomic and geospatial uncertainties/errors needed to be checked. It would be desirable to process these data to include them in Biodiversidata’s database in the future. For instance, georeferencing efforts could be implemented to increase the number of records to include (Chapman and Wieczorek 2020, Zermoglio et al. 2020).

A single dataset with 5,138 occurrence records was downloaded, available at: https://doi.org/10.15468/dl.wc2fm7. After the data cleaning and quality check process was performed (see details in Quality control subsection), we kept 3,428 data records. Of those records, 1,787 corresponded to specimens and were contributed to GBIF by 51 different institutions around the world. The major contributor was the Missouri Botanical Garden (28.8% of the 1,787 records), followed by Universidade Federale do Rio Grande do Sul of Brazil (11.8%) and Universidade de São Paulo (6.6%). The 1,637 human observations were mainly derived (99.6%) from the citizen-science platform iNaturalist.

The data provided by Biodiversidata members were curated (e.g. taxonomic names updated, fields standardised) and uploaded to GBIF as four separate datasets, one for each data contributor (see sources in Table 2). These records were mostly part of research project surveys, 77.5% of them being observations and 22.5% have a specimen deposited in national natural history collections, such as the Herbarium of the Museo Nacional de Historia Natural de Uruguay (MVM) and the Museo y Jardín Botánico Prof. Atilio Lombardo (MVJB).

### Quality control

For data to be fit for use, they must be accurate, complete, consistent with other sources and provide a proper level of detail (Chapman 2005). To meet these standards, we performed the subsequent steps for all the data (see R scripts and working examples at github.com/bienflorencia/rBiodiversidata):

### Step description

We checked misspellings, format errors and resolved synonymy and we completed higher taxonomic and infraspecific ranks terms and taxonomic authority for the scientific names using the R packages 'taxize' (Chamberlain et al. 2020b) and ‘WorldFlora’ (Kindt 2020). To check and unify species scientific names, for simplicity we first contrasted the list of species names to World Flora Online (WFO) Taxonomic Backbone. For species derived from literature and Biodiversidata members sources, we used verbatim species names against the authority sources and for GBIF data, we used the 'scientificName' field. If the species match were accepted by Zuloaga et al. (2019) in Darwinion, we kept the name and taxon ID of WFO, otherwise, we used the accepted name from Darwinion and searched for a taxon ID in Tropicos. The original species name was kept under the term ‘previousIdentification’. Additionally, the term ‘establishmentMeans’ was added, categorising species as native or introduced (in Spanish: *nativa* and *introducida*) according to Andrade et al. (2018). Species with unverified occurrence in the country were excluded. The final species list was checked by the Biodiversidata plant experts.

We checked dates accuracy and completed the 'eventDate' term with the format YYYY-MM-DD (e.g. 2020-02-10 for 20 February 2010). If only the year were known, 'eventDate' was represented as YYYY and if only the year and month were known, as YYYY-MM.

We filtered records occurring outside Uruguay's continental territory and checked for inaccuracy and incompleteness in georeferences. The data accessed via GBIF was filtered by keeping records with coordinate uncertainty values of less than 10 km and discarding those records with country centroid as georeference protocol. This hard filter was performed to reduce processing time and avoid location inaccuracy for posterior analyses. For the data extracted from literature, when coordinates were missing, we georeferenced point localities from maps figures using Google Earth Pro 2020 and marked them as requiring further verification. From the data provided by members of Biodiversidata or collated from literature, when geographic coordinates were presented either as degrees, minutes and seconds or degrees and decimal minutes, we georeferenced the locations to decimal degrees, following georeferencing best practices (Chapman and Wieczorek 2020, Zermoglio et al. 2020), including datum, uncertainty, precision, georeferencing protocol and georeferencing date values for all these records. Finally, we included the higher geography terms 'continent' and 'country' and the 'stateProvince' term for all the records in the database through the GeoNames Gazetteer database using the R package ‘geonames’ (Rowlingson 2019).

Finally, we generated a unique 'occurrenceID' for every record in our database, except the data accessed from GBIF for which we kept the original ID.

## Geographic coverage

### Description

**Figure 1.**
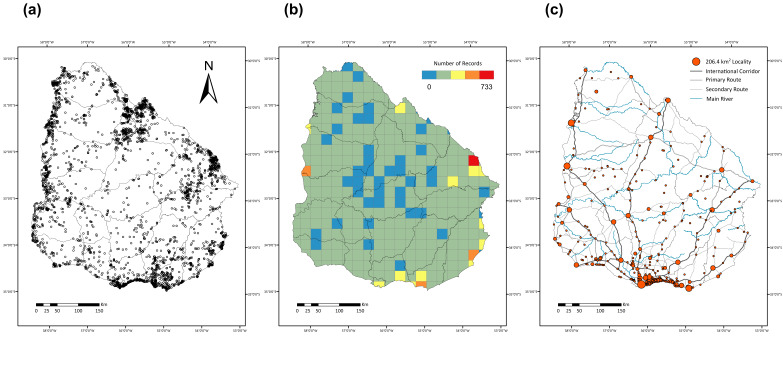
Distribution in Uruguay of (a) the total 12,470 occurrence records of vascular plants in Biodiversidata, (b) sampling effort with 25 × 25 km grid-cell resolution (the mid-size resolution used for Biodiversidata's analyses) and (c) urban areas (orange dots with size relative to surface in km^2^), routes (international, primary and secondary) and main rivers. Projection WGS1984.

The database covers extant species of vascular plants reported for locations within the borders of Uruguay. The occurrence records are spatially biased (Fig. 1a), as larger numbers of records are restricted to areas around the borders of the country, whilst the central regions of the territory have lower levels of sampling. The most sampled area of Uruguay is in Cerro Largo (central-eastern part of the country, at the frontier with Brazil), followed by the surroundings of some cities on the Atlantic coast (Fig. 1b). As previously observed in tetrapods (Grattarola et al. 2019a) and woody flora (Haretche et al. 2012), some areas of the country remain systematically neglected. It is currently unclear whether these disparities in sampling are due to the lack of explorations, the lack of digitisation or georeferencing of existing occurrences (e.g. GBIF records discarded in the preparation of the database), given the high taxonomic complexity of some vascular plant families for records to reach species level or a combination of all the above. Additionally, the lack of explorations could be for multiple reasons: either a result of the difficulty to access certain areas (see in Fig. 1c, the distribution of urban areas, main routes and rivers) or because of the preference of botanists for certain landscapes over others (Haretche et al. 2012). Maps were created in R and figures prepared using ArcGis 10.5. Sampling effort was evaluated as the number of records in each cell (see scripts in github.com/bienflorencia/rBiodiversidata for a working example). For the Biodiversidata project, Uruguay’s territory is divided in grid-cells of three different sizes: 50 x 50, 25 x 25 and 12.5 x 12.5 km; here we present sampling effort values with the mid-size unit of 25 km.

### Coordinates

-58.43882 and -53.266525 Latitude; -30.10818 and -34.973188 Longitude.

## Taxonomic coverage

### Description

**Figure 2.**
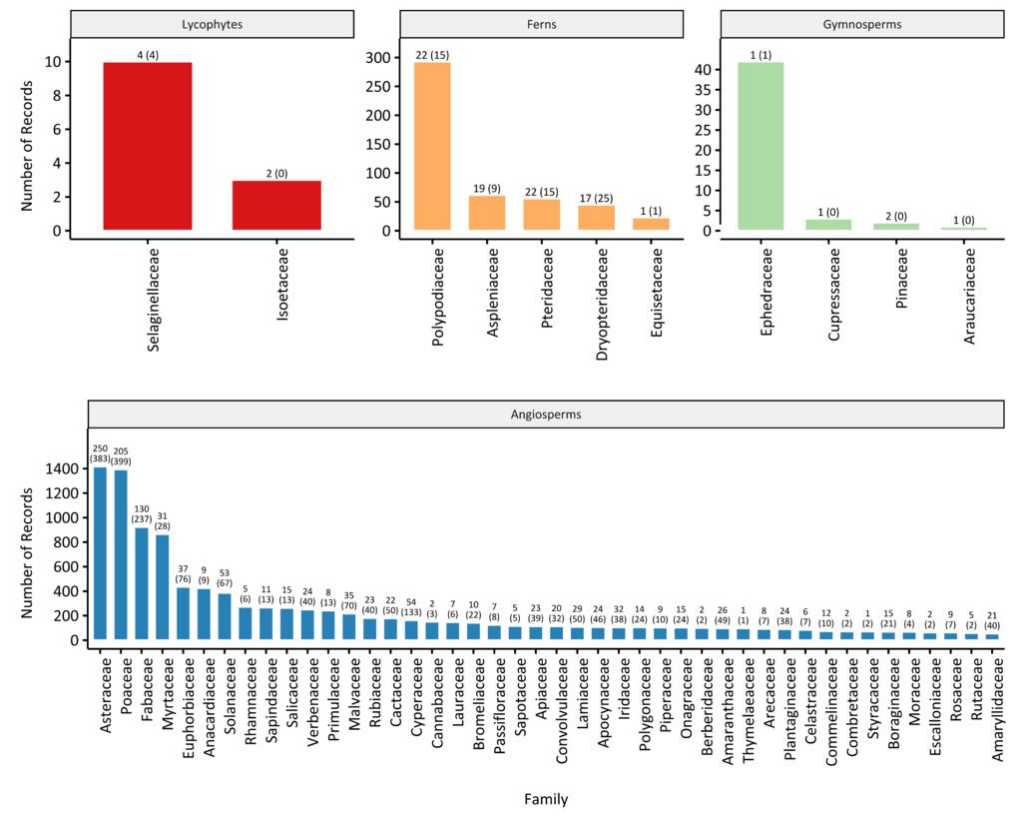
Number of occurrence records of vascular plants from Uruguay per family within each clade, in Biodiversidata. For Ferns and Angiosperms, only families with more than 20 and 50 records, respectively, are shown. On top of the bars, the number of species for each family is included along with the corresponding number of species that is expected by Andrade et al. (2018) between brackets.

The database includes 1,362 native species, 271 introduced and 15 species of yet unknown establishment means. According to Andrade et al. (2018), in Uruguay there are 167 families of vascular plants, comprising 2,431 native species. Therefore, our database covers 56.1% of the native species and 94.7% of the families that have been recorded in the country. The taxonomic coverage amongst groups is uneven (Fig. 2) fairly reflecting the current richness dominance of some taxa groups over others. See on top of the bars in Fig. 2 the number of species in the database and those expected by Andrade et al. (2018) for Uruguay within each family. Families with the greatest number of species in our database are Asteraceae (N = 250), Poaceae (N = 205), Fabaceae (N = 130) and Cyperaceae (N = 54).

### Taxa included

**Table:** 

Rank	Scientific Name	Common Name
kingdom	Plantae	Plants
phylum	Tracheophyta	Vascular Plants

## Temporal coverage

**Data range:** 1877-2-01 – 2020-5-21.

### Notes

**Figure 3.**
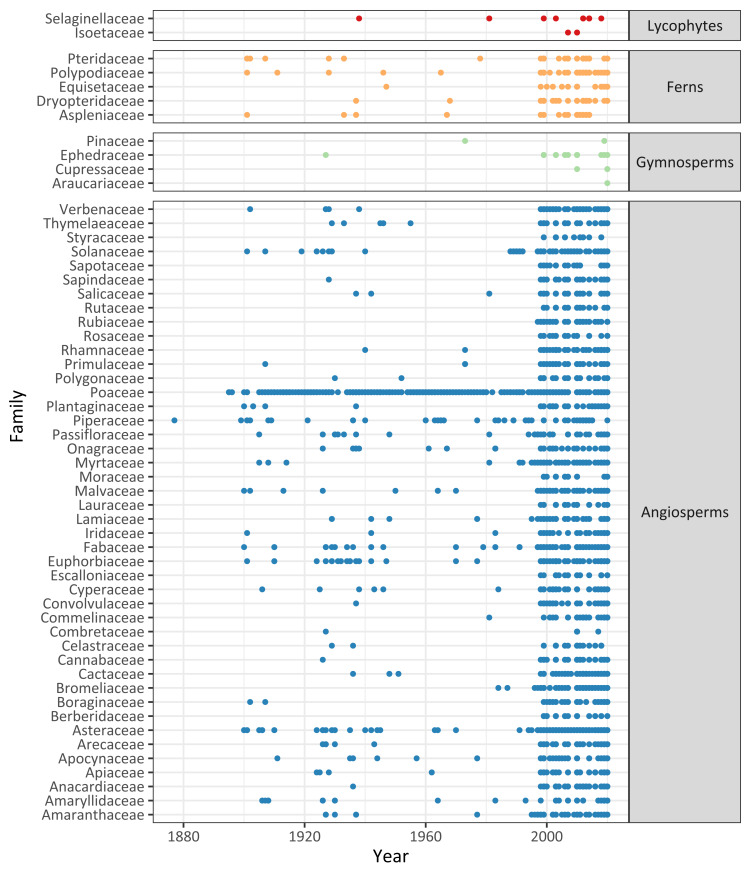
Representation of vascular plants families from Uruguay over time, grouped by clades, in Biodiversidata. Dots indicate there is at least one occurrence record for a species in the given family. For Ferns and Angiosperms, only families with more than 20 and 50 records, respectively, are shown.

The records included in the database cover samples reported in Uruguay during the period of 1877–2020 (Fig. 3). A large proportion of the records has information about the date of collection/observation (89.2%). We observed that occurrence records have been collected mostly irregularly within groups, with some families, such as Poaceae and Piperaceae, represented in larger time periods, yet most exclusively or more intensely in the last 20-30 years.

## Usage licence

### Usage licence

Other

### IP rights notes

Creative Commons Attribution License (CC-BY 4.0)

## Data resources

### Data package title

Biodiversidata

### Resource link

https://doi.org/10.5281/zenodo.3954406

### Number of data sets

1

### Data set 1.

#### Data set name

Biodiversidata: Vascular Plant Species Occurrences of Uruguay

#### Data format

Darwin Core occurrence data CSV

#### Number of columns

61

#### Download URL

http://doi.org/10.5281/zenodo.3954406

#### Data format version

1.0.0

#### Description

The dataset provides primary biodiversity data on extant vascular plant species recorded within Uruguay between 1877–2020 (Suppl. material 1). The total number of records collated is 12,470, including 1,648 species and 61 columns of Darwin Core standard terms (descriptions adapted from https://dwc.tdwg.org/terms/).

**Data set 1.** 

Column label	Column description
occurrenceID	An identifier for the existence of a particular organism at a particular place at a particular time | dwc:occurrenceID
otherCatalogNumbers	A list (concatenated and separated) of previous or alternate fully qualified catalogue numbers or other human-used identifiers for the same particular organism, whether in the current or any other dataset or collection | dwc:otherCatalogNumbers
basisOfRecord	The specific nature of the data record (e.g. PreservedSpecimen, HumanObservation, unknown) | dwc:basisOfRecord
recordedBy	A list (concatenated and separated) of names of people, groups or organisations responsible for recording the original existence of a particular organism at a particular place at a particular time | dwc:recordedBy
establishmentMeans	The process by which the biological individual(s) represented in the record established at the spatial region or named place (e.g. native (= nativa), introduced (= introducida), unknown (= desconocido)) | dwc:establishmentMeans
previousIdentifications	A list (concatenated and separated) of previous assignments of names to the recorded organism | dwc:previousIdentifications
eventDate	The date during which the recording event occurred (format YYYY-MM-DD) | dwc:eventDate
year	The four-digit year in which the recording event occurred, according to the Common Era Calendar | dwc:year
month	The ordinal month in which the recording event occurred | dwc:month
day	The integer day of the month on which the recording event occurred | dwc:day
higherGeography	A list (concatenated and separated) of geographic names less specific than the information captured in the locality term | dwc:higherGeography
continent	The name of the continent in which the spatial region or named place occurs | dwc:continent
country	The name of the country or major administrative unit in which the spatial region or named place occurs | dwc:country
countryCode	The standard code for the country in which the spatial region or named place occurs | dwc:countryCode
stateProvince	The name of the next smaller administrative region than country (state, province, canton, department, region etc.) in which the location occurs | dwc:stateProvince
locality	The standardised description of the spatial region or named place of an event | dwc:locality
decimalLatitude	The geographic latitude (in decimal degrees, using the spatial reference system given in geodeticDatum) of the geographic centre of a spatial region or named place | dwc:decimalLatitude
decimalLongitude	The geographic longitude (in decimal degrees, using the spatial reference system given in geodeticDatum) of the geographic centre of a spatial region or named place | dwc:decimalLongitude
geodeticDatum	The ellipsoid, geodetic datum, or spatial reference system (SRS) upon which the geographic coordinates given in decimalLatitude and decimalLongitude as based | dwc:geodeticDatum
coordinateUncertaintyInMeters	The horizontal distance (in metres) from the given decimalLatitude and decimalLongitude describing the smallest circle containing the whole of the spatial region or named place | dwc:coordinateUncertaintyInMeters
coordinatePrecision	A decimal representation of the precision of the coordinates given in the decimalLatitude and decimalLongitude | dwc:coordinatePrecision
georeferencedBy	A list (concatenated and separated) of names of people, groups or organisations who determined the georeference (spatial representation) for the spatial region or named place | dwc:georeferencedBy
identifiedBy	A list (concatenated and separated) of names of people, groups or organisations who assigned the taxon to the subject | dwc:identifiedBy
taxonID	An global unique identifier for the set of taxon information | dwc:taxonID
scientificName	The full scientific name, with authorship and date information if known | dwc:scientificName
nameAccordingTo	The reference to the source in which the specific taxon concept circumscription is defined or implied | dwc:nameAccordingTo
higherClassification	A list (concatenated and separated) of taxa names terminating at the rank immediately superior to the taxon referenced in the taxon record | dwc:higherClassification
kingdom	The full scientific name of the kingdom in which the taxon is classified | dwc:kingdom
phylum	The full scientific name of the phylum or division in which the taxon is classified | dwc:phylum
class	The full scientific name of the class in which the taxon is classified | dwc:class
order	The full scientific name of the order in which the taxon is classified | dwc:order
family	The full scientific name of the family in which the taxon is classified | dwc:family
genus	The full scientific name of the genus in which the taxon is classified | dwc:genus
specificEpithet	The name of the first or species epithet of the scientificName | dwc:specificEpithet
infraspecificEpithet	The name of the lowest or terminal infraspecific epithet of the scientificName, excluding any rank designation | dwc:infraspecificEpithet
taxonRank	The taxonomic rank of the most specific name in the scientificName | dwc:taxonRank
scientificNameAuthorship	The authorship information for the scientificName | dwc:scientificNameAuthorship
institutionCode	The name (or acronym) in use by the institution having custody of the object(s) or information referred to in the record | dwc:institutionCode
collectionCode	The name, acronym, coden or initialism identifying the collection or dataset from which the record was derived | dwc:collectionCode
catalogNumber	An identifier (preferably unique) for the record within the dataset or collection | dwc:catalogNumber
recordNumber	An identifier given to the occurrence at the time it was recorded | dwc:recordNumber
associatedReferences	A list (concatenated and separated) of identifiers (publication, bibliographic reference, URI) of literature associated with the existence of a particular organism at a particular place at a particular time | dwc:associatedReferences
verbatimLocality	The original textual description of the spatial region or named place of the record event | dwc:verbatimLocality
georeferenceRemarks	Notes or comments about the spatial description determination, explaining assumptions made | dwc:georeferenceRemarks
vernacularName	A list (concatenated and separated) of common or vernacular names | dwc:vernacularName
locationAccordingTo	Information about the source of the location information | dwc:locationAccordingTo
georeferencedDate	The date on which the location was georeferenced | dwc:georeferencedDate
georeferenceSources	A list (concatenated and separated) of maps, gazetteers or other resources used to georeference the Location, described specifically enough to allow anyone in the future to use the same resources | dwc:georeferenceSources
georeferenceVerificationStatus	A categorical description of the extent to which the georeference has been verified to represent the best possible spatial description (e.g. requires verification) | dwc:georeferenceVerificationStatus
georeferenceProtocol	A description or reference to the methods used to determine the spatial coordinates and uncertainties | dwc:georeferenceProtocol
verbatimLatitude	The verbatim original latitude of the location (spatial region or named place) | dwc:verbatimLatitude
verbatimLongitude	The verbatim original longitude of the location (spatial region or named place) | dwc:verbatimLongitude
verbatimCoordinateSystem	The spatial coordinate system for the verbatimLatitude and verbatimLongitude of the location | dwc:verbatimCoordinateSystem
locationRemarks	Comments or notes about the location (spatial region or named place) | dwc:locationRemarks
measurementType	The nature of the measurement, fact, characteristic or assertion | dwc:measurementType
measurementValue	The value of the measurement, fact, characteristic or assertion | dwc:measurementValue
measurementDeterminedBy	A list (concatenated and separated) of names of people, groups or organisations who determined the value of the measurement, fact, characteristic or assertion | dwc:measurementDeterminedBy
measurementRemarks	Comments or notes accompanying the measurement, fact, characteristic or assertion | dwc:measurementRemarks
organismRemarks	Comments or notes about the particular organism recorded | dwc:organismRemarks
language	Language of the resource | dcterms:language
license	A legal document giving official permission to do something with the resource | dcterms:license

## Additional information

Biodiversidata is a collaborative association of experts with the aim of assembling a constantly-growing database for Uruguay’s biodiversity. The initiative was launched in 2018 under the direction of Florencia Grattarola as part of her PhD project at the University of Lincoln in partnership with the MacroBiodiversity Lab at Queen’s University Belfast (UK), led by Daniel Pincheira-Donoso. Its open-access platform (https://biodiversidata.org/) aims to make the biodiversity data of Uruguay openly available by integrating a broad range of resources including databases, publications, maps, reports and infographics, derived from the work of the team of expert scientific members. Current funds for developing Biodiversidata are conditional upon Grattarola's PhD project concluding in December 2020. The database presented in this study will continue to be improved and updated with new records periodically (yearly expected); check the Zenodo repository for the latest version: doi.org/10.5281/zenodo.3954406

### Members of the Biodiversidata Consortium

Florencia Grattarola, Germán Botto, Laura Capuccio, Inés da Rosa, César Fagúndez, Noelia Gobel, Andrés González, Enrique M. González, Javier González, Daniel Hernández, Gabriel Laufer, Patricia Mai, Raúl Maneyro, María Martínez, Juan A. Martínez-Lanfranco, Daniel E. Naya, Ana L. Rodales, Florencia Rossi, Franco Teixeira de Mello, Lucía Urtado, Lucía Ziegler and Daniel Pincheira-Donoso.

## Supplementary Material

92E4F74F-C570-5332-87C4-FD947BC4FA2F 10.3897/BDJ.8.e56850.suppl1 Supplementary material 1 Biodiversidata vascular plant occurrence records from Uruguay Data type primary biodiversity data (occurrences) Brief description Comma-separated csv data file containing the 12,470 species occurrence records held in the Biodiversidata database by 2020-08-28 File: oo_447351.csv https://binary.pensoft.net/file/447351 Florencia Grattarola, Andrés González, Patricia Mai, Laura Cappuccio, César Fagúndez-Pachón, Florencia Rossi, Franco Teixeira de Mello, Lucía Urtado, Daniel Pincheira-Donoso

## Figures and Tables

**Table 1.** Records collected per group showing number of occurrence records, number of species, records with date of collection, records collected in the last 30 years and records with coordinates, with percentage in parentheses.

**Group**	**Number of Occurrence Records**	**Number of Species**	**Records with Date (%)**	**Records from the last 30 years (%)**	**Records with Coordinates (%)**
Lycophytes	13	6	13 (100)	11 (84.6)	13 (100)
Ferns	540	78	540 (100)	508 (94.1)	540 (100)
Gymnosperms	48	5	41 (85.4)	39 (81.2)	48 (100)
Angiosperms	11,869	1,559	10,527 (88.7)	9,585 (80.8)	11,869 (100)
Total	12,470	1,648	11,121 (89.2)	10,143 (81.3)	12,470 (100)

**Table 2.** List of sources used to build the Biodiversidata plant dataset, including the source type, the plant groups included in each source and the number of records extracted from each of the sources.

**Source**	**Source Type**	**Groups**	**Records**
Alonso Paz and Bassagoda (1999)	Journal Article	Ferns, Gymnosperms, Angiosperms	252
Alonso Paz and Bassagoda (2003)	Journal Article	Ferns, Gymnosperms, Angiosperms	107
Alonso Paz and Bassagoda (2009)	Journal Article	Angiosperms	3
Bartesaghi (2007)	Thesis	Gymnosperms, Angiosperms	34
Berazategui et al. (2009)	Short Communication	Angiosperms	2
Berazategui et al. (2010)	Short Communication	Angiosperms	2
Berazategui et al. (2012)	Short Communication	Angiosperms	3
Brussa (2016)	Journal Article	Angiosperms	8
Cascales et al. (2014)	Journal Article	Angiosperms	6
Delfino and Masciadri (2005)	Journal Article	Ferns, Angiosperms	153
Delfino et al. (2005)	Journal Article	Angiosperms	26
Etchebarne (2014)	Thesis	Angiosperms	71
Fagúndez-Pachón and Grattarola (2020)	Biodiversidata member	Lycophytes, Ferns, Gymnosperms, Angiosperms	340
Gautreau and Lezama (2009)	Journal Article	Angiosperms	52
GBIF (2020)	Online Database	Lycophytes, Ferns, Gymnosperms, Angiosperms	3428
González and Grattarola (2020)	Biodiversidata member	Angiosperms	101
González-Cabezudo (2011)	Thesis	Angiosperms	781
González-Calcagno (2013)	Thesis	Angiosperms	991
Grela (2004)	Thesis	Gymnosperms, Angiosperms	1343
Grela and Brussa (2003)	Journal Article	Gymnosperms, Angiosperms	897
Guido and Mársico (2011)	Journal Article	Angiosperms	17
Guido et al. (2013)	Journal Article	Angiosperms	14
Jolochin (2007)	Thesis	Angiosperms	68
Jolochin (2016)	Thesis	Angiosperms	20
Machado and González-Rosales (2016)	Thesis	Lycophytes, Ferns, Angiosperms	220
Mai et al. (2020)	Biodiversidata member	Ferns, Angiosperms	520
Mai et al. (2016)	Journal Article	Angiosperms	50
Pérez-Sobrino (2016)	Thesis	Angiosperms	9
Ramos (2009)	Thesis	Angiosperms	152
Ríos et al. (2010)	Report	Lycophytes, Ferns, Gymnosperms, Angiosperms	1357
Rivas and Barilani (2004)	Journal Article	Angiosperms	2
Rivas et al. (2014)	Journal Article	Ferns, Angiosperms	53
Rivas et al. (2017)	Journal Article	Lycophytes, Ferns, Gymnosperms, Angiosperms	283
Rodríguez-Mazzini et al. (2001)	Report	Lycophytes, Ferns, Gymnosperms, Angiosperms	710
Rossado et al. (2018)	Journal Article	Angiosperms	20
Trujillo (2012)	Thesis	Angiosperms	9
Urtado et al. (2020)	Biodiversidata member	Angiosperms, Ferns	366
